# The Impact of Postoperative Complications on Long-Term Oncological Outcomes Following Curative Resection of Colorectal Cancer (Stage I-III): A Systematic Review and Meta-Analysis

**DOI:** 10.7759/cureus.12837

**Published:** 2021-01-21

**Authors:** Noor M Mualla, Maryam R Hussain, Muhammad Akrmah, Preeti Malik, Sadia Bashir, Jenny J Lin

**Affiliations:** 1 Public Health, Icahn School of Medicine at Mount Sinai, New York, USA; 2 Epidemiology, Icahn School of Medicine at Mount Sinai, New York, USA; 3 Pathology, Hartford Hospital, Hartford, USA; 4 Neurology, Massachusetts General Hospital, Andover, USA; 5 Internal Medicine, Pakistan Medical and Dental Council and University of Health Sciences, Lahore, PAK; 6 Internal Medicine, Icahn School of Medicine at Mount Sinai, New York, USA

**Keywords:** post-operative complications, colorectal cancer, overall survival, disease free survival, overall recurrence

## Abstract

Background and objective

The incidence of post-surgical complications (PSCs) after curative resection of non-metastatic colorectal cancer (CRC) is very widespread and evident. Some studies suggest that PSCs often predict poor long-term oncological outcomes. However, the available data on the topic is often controversial. The aim of this systematic review and meta-analysis was to study the effect of postoperative complications (POCs) on long-term oncological outcomes following curative resection of non-metastatic (stage I-III) CRC.

Methods

A comprehensive search of MEDLINE® and Excerpta Medica dataBASE (EMBASE) databases was performed via the Ovid platform, by using controlled vocabulary as well as natural language terms for POCs, outcomes, and CRC. Two authors independently screened the studies and extracted data. Conflicts were resolved by discussion among authors and also independently with the help of a third author. Meta-analysis was performed using a random-effects model (REM) to calculate pooled estimates for overall survival (OS), disease-free survival (DFS), and overall recurrence.

Results

Overall, 3,836 studies were initially screened, and 16 studies involving 37,192 patients were ultimately selected for final inclusion in the analysis. Meta-analysis of these studies showed that PSCs following non-metastatic CRC surgery predicted worse OS rates [hazard ratio (HR): 1.36; 95% CI: 1.15-1.61; p<0.00001], DFS (HR: 1.41; 95% CI: 1.11-1.80; p<0.00001), and overall recurrence (HR: 1.19; 95% CI: 1.04-1.37; p=0.01).

Conclusion

Based on our findings, PSCs predict poor OS rates, DFS, and overall recurrence following curative resection of non-metastatic CRC.

## Introduction

Colorectal cancer (CRC) constitutes a significant burden on healthcare systems worldwide and is the third leading cause of mortality in the United States. According to the latest Centers for Disease Control and Prevention (CDC) data published in 2016, there were 52,286 deaths related to CRC annually [1]. Surgical resection continues to be the most preferred treatment modality for CRC but is often associated with significant postoperative complications (POCs) and morbidity [2]. POCs are associated with a prolonged hospital stay, increased hospital cost, and increased reoperation rates. POCs can be described as “deviation from the normal postoperative course”. Studies have reported different rates of POCs ranging between 10-37%, depending on the type and severity of complications and study design [3]. Furthermore, it has been increasingly seen in the literature that POCs following surgery for CRC can have a huge negative impact on both short- and long-term survival of the patients [3-18].

A study by Law et al. [8] has demonstrated that POCs, especially infection-related ones, have significantly negatively affected the overall survival (OS) and overall recurrence rate in stage I-III CRC patients after curative resection. However, the severity of the complications was not stratified by the authors. Odermatt et al. [11] have reported a reduced OS among patients with major complications following CRC resection, but the same negative influence was not observed for disease-free survival (DFS). Many studies have indicated that anastomotic leak is the most feared complication after colorectal surgery, and a meta-analysis of these studies have reported a significant negative impact of an anastomotic leak following colorectal surgery on local recurrence and reduced cancer-specific survival [19]. Artinyan et al. [5] analyzed 12,075 patients from a system-wide database of veterans in the United States and found that POCs, especially infectious complications after CRC resection, were associated with decreased OS rates independent of patient, treatment, and disease factors. However, this study had >90% male population from the database, lacked detailed information on the complications, and did not explore disease recurrence. Previous studies have reported significantly worse long-term outcomes in patients with POCs in various cancers including pancreatic cancer, gastric cancer, hepatocellular cancer, CRC, and metastatic liver tumors [8,20-22]. 

The studies in the literature mentioned above have demonstrated significantly reduced long-term survival in patients with complications after surgical resection for CRC. However, there are several limitations to these studies, with a majority of them being single-center studies and small-population studies. In addition, there are several studies showing evidence of less favorable long-term outcomes in patients with POCs after CRC surgery with liver metastasis [23,24]. Furthermore, studies evaluating the impact of complications after curative resection for stage I-III CRC patients are scarce [3].

Hence, the aim of this systematic review was to examine the relationship between POCs and long-term outcomes such as OS, DFS, and recurrence rate following surgery for stage I-III CRC.

## Materials and methods

This meta-analysis adhered to the recommendations of the Preferred Reporting Items for Systematic Reviews and Meta-Analyses (PRISMA) statement [25]. This systematic review of published literature was conducted to assess the impact of PSCs on OS, DFS, and recurrence rates following surgery for stage I-III CRC.

Data and literature search

A comprehensive search of the literature was conducted using the US National Library of Medicine (MEDLINE®) and Excerpta Medica dataBASE (EMBASE) via the Ovid platform from inception to December 2019. The following search strategy was employed: (1) exp Postoperative Complications/ (2) Postoperative complication*. ti, ab,kf. (3) exp Rectal Neoplasms/ (4) exp Colorectal Neoplasms/ (5) exp Colonic Neoplasms/ (6) exp Sigmoid Neoplasms/ (7) exp Anus Neoplasms/ (8) (colorectal cancer or colon cancer or rectal cancer or sigmoid cancer).ti,ab,kf. (9) (Perioperative Complication* or Postoperative* Complication).ti,ab,kf. (10) exp Mortality/ (11) exp Survival/ (12) exp Recurrence/ or exp Neoplasm Recurrence, Local/, (13) exp "Quality of Life"/ (14) (Recurrence or Survival or risk or mortality or outcome* or "quality of life").ti,ab,kf. (15) Perioperative risk.ti,ab,kf. (16) 1 or 2 or 9 or 15 (17) 3 or 4 or 5 or 6 or 7 or 8 (18) 10 or 11 or 12 or 13 or 14 (19) 16 and 17 and 18. The search terms were finalized after multiple pilot searches using more inclusive terms were run, which returned large numbers of abstracts that, on initial assessment, were found to be irrelevant to the present review topic.

Inclusion and data extraction

The titles and abstracts of all returned studies were independently examined by two authors (N.M. and M.H.) for relevance. Review articles, non-English papers, animal studies, and conference proceedings with abstract-only results were excluded. Full texts of only potentially relevant studies were acquired and analyzed. To be included in the meta-analysis, studies had to report the influence of POCs on OS or DFS or recurrence following surgery for stage I-III CRC. In case of disagreement or confusion over the eligibility of a study, two additional reviewers (M.A. and P.M.) assessed the article until a consensus was reached.

Reviewers (N.M. and M.H.) also independently extracted the data from eligible studies including first author and year of publication, the country where the study was conducted, study design, patient characteristics including sex and age, the total number of subjects, the incidence of complications, sites of tumors, and staging of cancer and long-term oncological outcomes including OS, DFS, and recurrence. Extracted data was cross-checked (M.A and P.M) to reach a consensus and entered into an Excel spreadsheet for analysis. Reference lists of included papers were manually searched for additional relevant studies.

Data analysis

Review Manager version 5.3 (The Nordic Cochrane Centre, The Cochrane Collaboration, Copenhagen, Denmark) was used for conducting the meta-analysis. The DerSimonian-Laird random-effects model (REM) was used to pool hazard ratios (HRs) for each outcome, accounting for heterogeneity in methodology and reporting of complications. The pooled HR and 95% CI are presented in the form of forest plots. Each square on the chart area represents an individual study and the area of each square is equivalent to the weight of the study, which is the inverse of the study variance. The diamond represents the summary measures and the width corresponds to the 95% CI.

The Z-test was employed to assess the overall impact of POCs on long-term oncological outcomes, and the heterogeneity was assessed by the I^2^ test. Two-tailed p-values of <0.05 were considered statistically significant. Publication bias was assessed visually with funnel plots. Sensitivity analysis was performed to assess the effect of publication bias and heterogeneity by excluding outlying studies on the funnel plot.

Assessment of study quality

The Newcastle-Ottawa Scale (NOS) was used to assess the quality and bias in the included studies, which rates selection, comparability, and outcome. All studies were determined to be of high quality.

## Results

Characteristics of studies and patients

**Figure 1:**
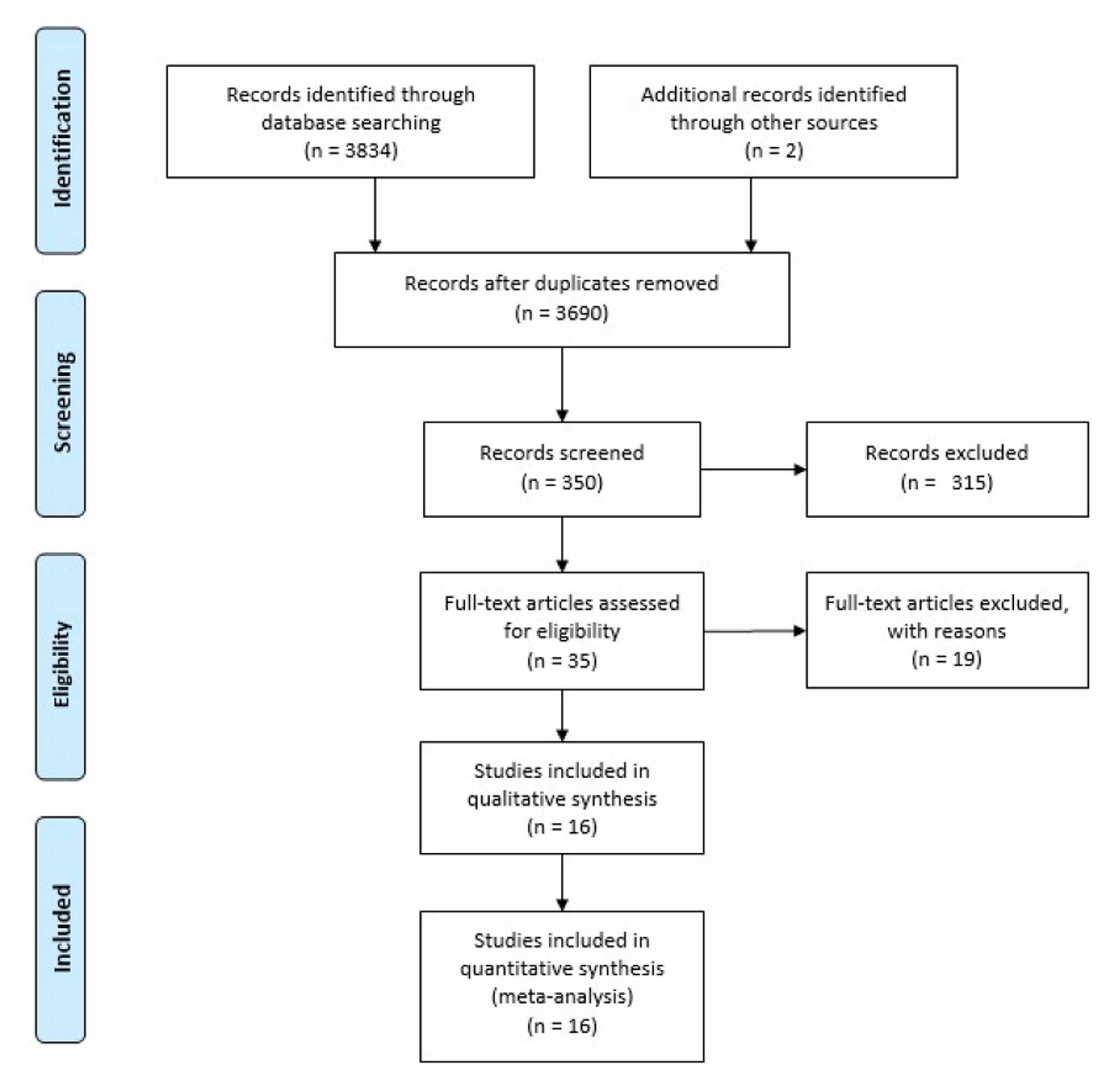
PRISMA flow diagram showing the selection and screening process of studies PRISMA: Preferred Reporting Items for Systematic Reviews and Meta-Analyses

A review of the databases initially identified 3,836 potentially relevant articles; 146 duplicated articles and 3,340 irrelevant articles were excluded during the first round of review, which only involved the titles. During the second round, abstracts of 350 articles were reviewed and 315 irrelevant articles were excluded. Full-text reviews of the remaining 35 articles were conducted, of which 19 articles were excluded with reasons specified. Hence, 16 studies were included in the final analysis (Figure 1), out of which 14 studies reported data for OS, 12 studies for DFS, and four studies for recurrence.

The 16 studies involved a total of 37,192 patients of which 69% were male and 31% were females; the median age of the patients was 65.91 years. Of note, 75% of patients had CRC and 25% had rectal cancer; 41% were in stages I and II and 21.5% were in stage III, and 24% developed POCs. Table 1 and Table 2 summarize the clinical characteristics of patients in all 16 included studies.

**Table 1:** Summary of studies about postoperative complications and long-term oncological outcomes including overall survival, disease-free survival, and recurrence RCS: retrospective cohort study; PCS: prospective cohort study; RCT: randomized controlled trial; OS: overall survival; DFS: disease-free survival; NR: not reported; HR: hazard ratio; CI: confidence interval

Study and year	Country	Study type	Number of patients (N=37,192)	Outcomes		
				OS	DFS	Recurrence
Law et al., 2007 [8]	Hong Kong	RCS	1,657	HR: 1.26; 95% CI: 1.03–1.52; p=0.023	NR	HR: 1.26; 95% CI: 1.01–1.57; p=.04
Richards et al., 2011 [13]	UK	RCS	423	HR: 1.36; 95% CI: 1.01–1.82; p=0.044	HR: 1.25; 95% CI: 0.89–1.77; p=0.197	NR
Mrak et al., 2013 [12]	Austria	RCS	811	HR: 0.86; 95% CI: 0.58–1.28; p=0.4556	HR: 0.96; 95% CI: 0.62–1.48; p=0.8392	NR
Tevis et al., 2013 [15]	USA	RCS	355	HR: 2.52; 95% CI: 1.25–5.03; p=0.009	NR	NR
Xia et al., 2014 [16]	China	RCS	224	HR: 2.74; 95% CI: 1.51–4.95; p=0.001	HR: 4.25; 95% CI: 2.29–7.88; p<0.001	NR
Odermatt et al., 2015 [11]	UK	RCS	844	HR: 2.42; 95% CI: 1.41–4.14; p=0.0036	HR: 1.77; 95% CI: 1.05–2.99; p=0.048	HR: 1.29; 95% CI: 0.56–2.99; p=0.55
Artinyan et al., 2015 [5]	USA	RCS	12,075	HR: 1.24; 95% CI: 1.15–1.34; p<0.001	NR	NR
McSorley et al., 2016 [9]	UK	PCS	377	HR: 1.30; 95% CI: 0.93–1.81; p=0.127	HR: 1.51; 95% CI: 0.98–2.33; p=0.061	NR
Park et al., 2016 [10]	South Korea	PCS	686	NR	HR: 1.65; 95% CI: 1.12–2.44; p=0.012	NR
Slankamenac et al., 2017 [14]	Switzerland	RCS	284	HR: 1.42; 95% CI: 0.7–2.8; p=0.32	HR: 1.3; 95% CI: 0.7–2.4; p=0.42	NR
Duraes et al., 2018 [7]	USA	RCS	2,266	HR: 0.63; 95% CI: 0.52–0.76; p<0.001	HR: 0.64; 95% CI: 0.54–0.76; p<0.001	HR: 1.35; 95% CI: 1.02–1.80; p=0.037
Aoyama et al., 2017 [4]	Japan	Pooled RCT	5,530	HR: 1.31; 95% CI: 1.12–1.54; p=0.001	HR: 1.24; 95% CI: 1.08–1.42; p=0.003	NR
Cienfuegos et al., 2018 [6]	Spain	RCS	950	NR	HR: 2.24; 95% CI: 1.03–4.97; p=0.04	HR: 1.04; 95% CI: 0.48–2.26; p=0.914
Huang et al., 2018 [3]	Taiwan	RCS	3,666	HR: 1.70; 95% CI: 1.37–2.11; p=0.001	HR: 1.63; 95% CI: 1.34-1.97; p=0.001	NR
Nowakowski et al., 2018 [17]	Poland	PCS	265	HR: 2.83; 95% CI: 1.35–5.92; p=0.0058	NR	NR
Arnarson et al., 2019 [18]	Sweden	RCS	6,779	HR: 1.34; 95% CI: 1.13–1.59	HR: 1.37; 95% CI: 1.13–1.64	NR

**Table 2:** Summary of the demographic and clinical characteristics of patients in the included studies *Median; **median with range; ***mean M/F: male/female; C+R: colorectal; R: rectal; C: colon; O+L: open and laparoscopic; O: open; L: laparoscopic; NR: not reported

Study and year	Number of subjects	Age in years (median)	Sex (M/F)	Patients with complications	Site of tumor	Type of surgery	Follow-up (months)	Adjuvant therapy
Law et al., 2007 [8]	1,657	70	943/714	452	C+R	O+L	45.3*	Yes
Richards et al., 2011 [13]	423	NR	230/193	142	C+R	O	80 (37–158)**	Yes
Mrak et al., 2013 [12]	811	65	489/322	268	R	NR	61.2***	Yes
Tevis et al., 2013 [15]	355	60	206/149	107	R	O+R	43.8*	Yes
Xia et al., 2014 [16]	224	NR	108/116	43	C	L	60 (6–80)**	Yes
Odermatt et al., 2015 [11]	844	72	387/456	39	C+R	O+R	68.4 (50.4–86.4)**	Yes
Artinyan et al., 2015 [5]	12,075	68.8	11,827/248	3,364	C+R	NR	72*	Yes
McSorley et al., 2016 [9]	377	65	208/169	138	C+R	O+R	46 (24–86)**	Yes
Park et al., 2016 [10]	686	60	421/265	175	R	L	38 (2–118)**	Yes
Slankamenac et al., 2017 [14]	284	65	182/102	105	C+R	O+R	23.5 (8.4–35.8)**	Yes
Duraes et al., 2018 [7]	2,266	65	1,374/892	669	C+R	L	63.6***	Yes
Aoyama et al., 2017 [4]	5,530	60	3,104/2,426	861	C+R	NR	60*	Yes
Cienfuegos et al., 2018 [6]	950	66	579/371	51	C	L	40*	Yes
Huang et al., 2018 [3]	3,666	67	2,375/1,291	823	C+R	L	58.7***	NR
Nowakowski et al., 2018 [17]	265	65	138/127	78	C+R	L	45 (34–55)**	NR
Arnarson et al., 2019 [18]	6,779	74	3,267/3,484	1,634	C	NR	60*	Yes

Impact of postoperative complications on overall survival

**Figure 2:**
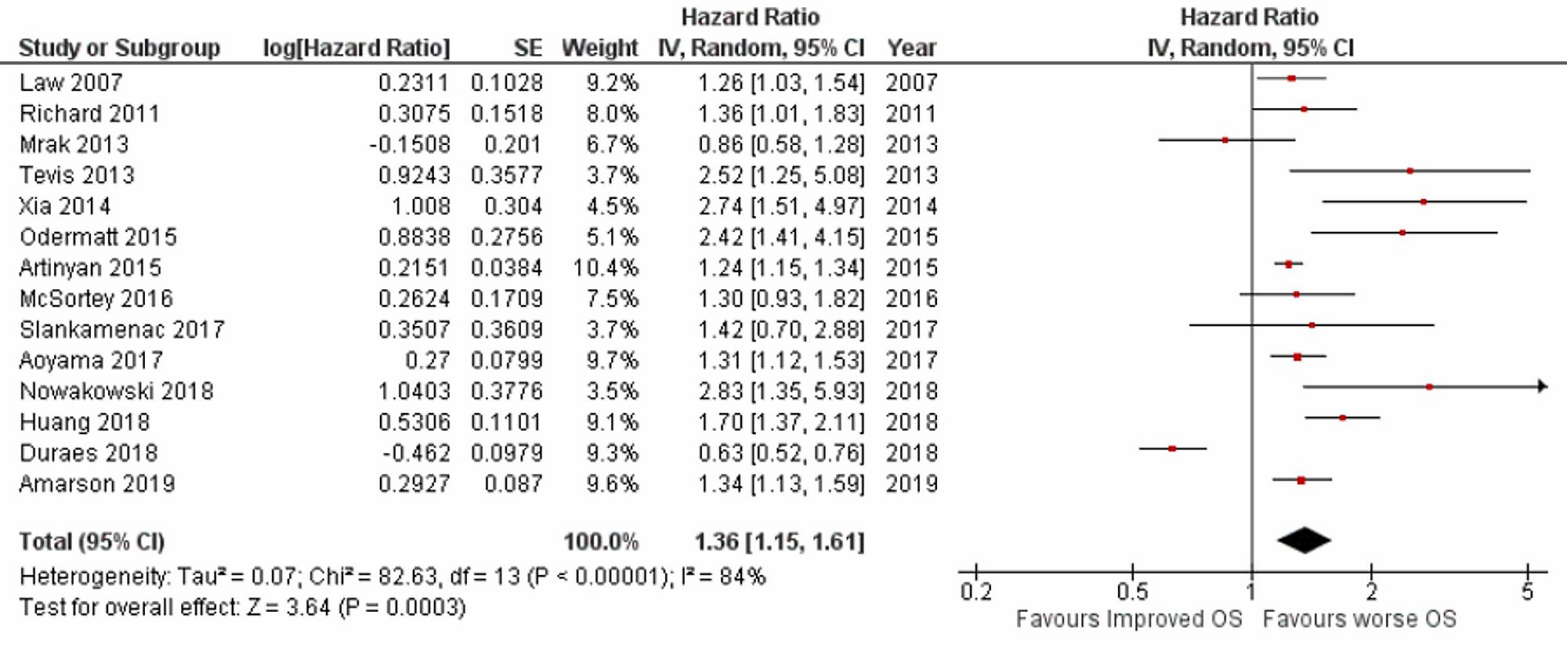
Forest plot showing the impact of postoperative complications on overall survival SE: standard error; IV: inverse variance; CI: confidence interval

Fourteen studies reported data on POCs and OS following curative resection of stage I-III CRC, giving a total sample size of 35,556 patients for evaluation. Out of these 14 studies, two studies [12,15] reported data solely for patients with rectal cancer, with a patient population of 1,166, and two studies [16,18] reported data for colon cancer, with a patient population of 7,003. A meta-analysis of all 14 studies showed that patients experiencing POCs had worse OS with a pooled HR of 1.36 (95% CI: 1.15-1.61; p=0.0003) (Figure 2). Between studies, heterogeneity was identified (p<0.00001; I^2^=84%), as well as possible publication bias on the funnel plot. Sensitivity analysis was conducted by eliminating the outlying studies on funnel plot in order to account for heterogeneity among the studies; results after sensitivity analysis still returned a significant pooled HR of 1.26 (95% CI: 1.19-1.33; p=0.0003) with no heterogeneity in the data (p=0.67; I^2^=0%).

Impact of postoperative complications on disease-free survival

**Figure 3:**
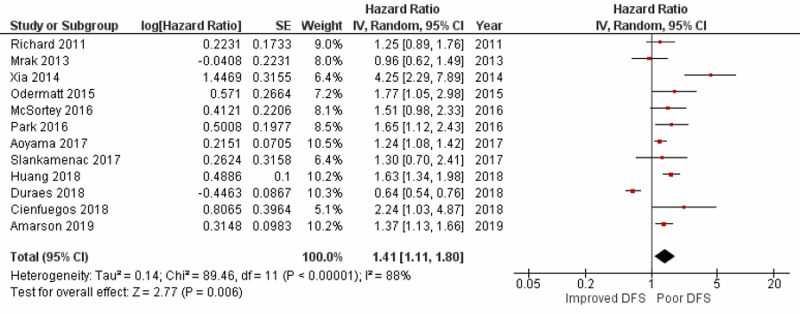
Forest plot showing the impact of postoperative complications on disease-free survival SE: standard error; IV: inverse variance; CI: confidence interval

A meta-analysis of 12 studies including 22,840 patients, reporting the impact of POCs on DFS following CRC, found a statistically significant worse DFS in patients with POCs in comparison to those who did not experience POCs, with a pooled HR of 1.41 (95% CI: 1.11-1.80; p=0.006) as shown in Figure 3. Study heterogeneity was found to be 88% (p<0.00001), which, after removing three outlying studies on sensitivity analysis, was reduced to 19% (p=0.28). Results after sensitivity analysis still showed a significant pooled HR of 1.37 (95% CI: 1.24-1.53; p=0.01).

Impact of postoperative complications on recurrence

**Figure 4:**
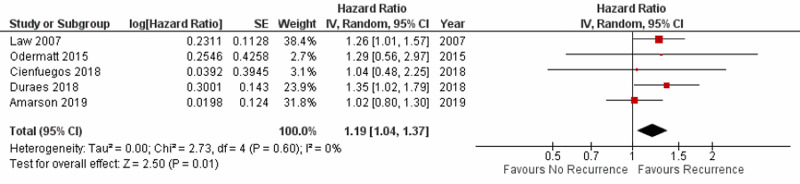
Forest plot showing the impact of postoperative complications on recurrence SE: standard error; IV: inverse variance; CI: confidence interval

Five studies reported data on the impact of POCs on overall recurrence after the resection of CRC. A meta-analysis of these five studies with a total patient population of 12,496 demonstrated that POCs predicted a higher overall recurrence of CRC with a pooled HR of 1.19 (95% CI:1.04-1.37; p=0.01) with 0% heterogeneity (p=0.60) as shown in Figure 4.

## Discussion

The results of our systematic review and meta-analysis showed that POCs have a significant harmful impact on long-term outcomes, including OS, DFS, and overall recurrence rate, in patients undergoing surgery for stage I-III CRC.

Sixteen studies with a combined population of 37,192 patients met the inclusion criteria and were included for our final analysis; 14 studies reported data for OS, 12 for DFS, and five for recurrence. Most of the studies reported combined data for colorectal surgeries and three studies [6,16,18] for colon and two for rectal [12,15] resection surgeries. The results showed that POCs have a clear and negative impact on OS and DFS. Fewer studies reported data for POCs and recurrence following CRC; however, a significant association was noted between patients faring poorly and POCs.

These results are consistent with other recent studies on the impact of POCs following surgery for CRC. A recent meta-analysis [26] of 18,611 patients from 14 studies reported that infectious POCs and complication severity had a significant negative impact on DFS (p=0.01, p<0.001) and OS (p<0.001, p<0.001) respectively. A study of 1,675 patients by Law et al. [8] demonstrated that POCs following curative resection in stage I-III CRC patients had a significantly worse OS (p=0.023), and a higher overall recurrence rate (p=0.04). In 2019, Arnason et al.'s [18] study of 6,779 patients undergoing resection for stage I-III colon cancer reported that both severe and non-severe POCs are significantly associated with decreased five-year OS and three-year DFS but not associated with increased recurrence rate. However, with respect to the type of complications, infective complications had similar effects on OS and DFS but a significant negative impact on recurrence rate.

To the best of our knowledge, there has been no other systematic review of the impact of POCs on long-term outcomes in stage I-III CRC patients to date. The results from the published literature taken together with the findings of the present meta-analysis indicate that POCs after surgery for stage I-III CRC have a significant adverse impact on long-term outcomes including OS, DFS, and recurrence rate.

The underlying mechanisms that link POCs with long-term oncological outcomes after curative resection are not clearly understood. One possible explanation is that trauma following the immediate postoperative period exaggerates the systemic inflammatory response (SIR), leading to increased markers such as serum C-reactive protein (CRP) and albumin and innate immune response, which in turn suppress cytotoxic immunity followed by triggering tumor progression and worsening the complications [27,28]. Furthermore, cancer cells have been identified in the bloodstream, bone marrow, and lymph nodes [29], and the release of these cancer cells together with SIR and immunosuppression may significantly influence OS and DFS in patients with POCs [12]. Hence, it is plausible that immunological modulation in SIRs following surgery might affect the spread of circulating tumor cells, adhesions development, thereby causing metastasis and consequently increasing the risk of recurrence. These findings have been reported in vitro by Tai et al. [30], and the results of the present review support this theory. However, it is unclear whether SIR is driven by type and severity of complications or if immune modulation by SIR is allowing the POCs to take over and cause poor long-term outcomes in patients with stage I-III CRC.

Strengths and limitations

One of the main limitations of the present systematic review is that we did not stratify the type and severity of complications and their effects on long-term outcomes; this was due to the limited number of published studies and available data. The majority of the studies were primarily observational studies, and only one Japanese study [4] provided pooled data from three randomized controlled trials. Studies were conducted mostly in large institutions often with a tertiary referral practice and using different criteria for diagnosing POCs and oncological outcomes. Another limitation is that the use of adjuvant chemotherapy was not reported for all included studies, which can potentially impact long-term survival. Additionally, heterogeneity was found among the meta-analyzed studies, which is why a decision was made to use REMs and post-hoc sensitivity analysis to exclude studies with wider 95% CIs and outlying effects. These measures may have significantly lessened the effects of observed heterogeneity, but it is unlikely that they were eliminated. Also, these results must be interpreted with caution as these findings of a statistically significant association between POCs and long-term oncological outcomes do not imply a causal association.

## Conclusions

The results of the present systematic review indicate that POCs have a significant adverse effect on long-term outcomes following surgery for stage I-III CRC. However, the underlying mechanism for such a finding is still not clear and can be the objective for future studies.
